# Psychological, Physiological, and Physical Effects of Resistance Training and Personalized Diet in Celiac Women

**DOI:** 10.3389/fnut.2022.838364

**Published:** 2022-06-16

**Authors:** Alejandro Martínez-Rodríguez, Daniela Alejandra Loaiza-Martínez, Javier Sánchez-Sánchez, Jacobo Á. Rubio-Arias, Fernando Alacid, Soledad Prats-Moya, María Martínez-Olcina, Rodrigo Yáñez-Sepúlveda, Nuria Asencio-Mas, Pablo J. Marcos-Pardo

**Affiliations:** ^1^Department of Analytical Chemistry, Nutrition and Food Science, Faculty of Sciences, University of Alicante, Alicante, Spain; ^2^Alicante Institute for Health and Biomedical Research (ISABIAL Foundation), Alicante, Spain; ^3^Faculty of Sports, Catholic University of Murcia (UCAM), Murcia, Spain; ^4^Universidad Tecnológica Indoamerica, Facultad de Ciencias de la Salud, Ambato, Ecuador; ^5^School of Sport and Science, European University of Madrid, Madrid, Spain; ^6^Department of Education, Health Research Centre, Faculty of Educational Sciences, University of Almería, Almería, Spain; ^7^Escuela de Educación, Pedagogía en Educación Física, Universidad Viña del Mar, Viña del Mar, Chile; ^8^SPORT Research Group (CTS-1024), CERNEP Research Center, University of Almería, Almería, Spain

**Keywords:** gluten free diet, physical activity, eating disorders, body composition, exercise

## Abstract

**Background:**

Gluten intolerance is a systemic process of autoimmune nature; it develops in genetically predisposed subjects with gluten ingestion. The only treatment for celiac disease (CD) is a lifelong strict gluten-free diet (GFD). This study was designed to evaluate adherence to a GFD, risk of an eating disorder, and nutritional status in adult CD patients undergoing different interventions.

**Methods:**

A total of 28 Spanish women, aged 40 years or more, took part in a randomized controlled trial. Each group received a different intervention: group 1, gluten-free nutrition plan + exercise (GFD + E); group 2, gluten-free nutrition plan (GFD); group 3, celiac controls (NO-GFD); and group 4, non-celiac controls (CONTROL). The training was prescribed by a sport scientist. It was based on resistance training with elastic bands; beforehand a warm-up was performed and the resistance was increased progressively. The variables studied were adherence to the GFD, risk of eating disorders, blood values, and body composition.

**Results:**

Celiac women with personalized nutritional planning presented greater adherence to a gluten-free diet (*p* < 0.001). Regarding leukocytes, significant differences were observed between the GFD and control groups (*p* = 0.004). Perimeters and folds did not decrease significantly.

**Conclusion:**

Women with celiac disease who follow an adapted and personalized diet have a better adherence to a GFD compared to those who follow a non-professional diet, and therefore have a better immune system status (blood leukocytes).

## Introduction

Gluten intolerance is a systematic process of autoimmune nature that develops in subjects with a genetic predisposition to the ingestion of gluten. It could appear at any age and remains throughout life (1). Celiac disease (CD) is a chronic disease, and it’s defined as a permanent gluten intolerance. Gluten is a protein present in some cereals such as wheat, barley, rye, triticale (a hybrid of wheat and rye), spelt, Kamut, and possibly oatmeal. In genetically predisposed individuals, this protein causes a serious injury to the small intestine mucosa, causing atrophy of the intestinal villi, which determines an inadequate absorption of nutrients from food (proteins, fats, sugars, carbohydrates, mineral salts, and vitamins), with the consequent clinical and functional repercussions (2).

The only treatment for CD is a strict gluten-free diet (GFD) for life. The adherence of the patients to the GFD is the key to a successful treatment and the prevention of additional complications, especially clinical manifestations (3). With this, the patients accomplish the disappearance of the symptoms, the normalization of the serology, and the recovery of the intestinal villi. On the other hand, failure to follow the diet can lead to important complications that can manifest themselves in the form of osteopenia, osteoporosis, and a high risk of neoplasm in the digestive tract, especially in adulthood (4–6). Some authors (6) suggest that due to this need for dietary management in CD, it can lead to the development of eating disorders. Eating behavior disorders are more common in chronically ill subjects compared to healthy individuals. From this fact, it seems that the presence of binge eating in people with CD may be related to excessive control over food.

In addition, it is quite common to observe a deficiency of some nutrients in patients, such as iron, calcium, zinc, folic acid, vitamin D, and other fat-soluble vitamins because of the malabsorption processes inherent to this pathology (3). Once patients follow a GFD, intestinal atrophy is initially restored, allowing for adequate nutrient absorption (7). However, some researchers have observed that celiac patients had an unbalanced diet in terms of macro- and micronutrients because of poor choices. Compared to healthy subjects, a higher consumption of simple sugars and fats has been observed in the total energy intake (8, 9). Regarding body composition, previous studies (10) have indicated that the prevalence of overweight women with celiac disease is low (6.5%), and there are very few cases of obesity (11).

In terms of physical activity, although there are several types of physical exercises, resistance training involves a type of exercise in which muscles are worked or maintained against an applied force. This is proposed as a first-line therapy to counteract age-related neuromuscular deterioration (12, 13). Specifically, elastic resistance (in the form of bands) is an alternative to traditional training devices that reduces the risk of injury, is accessible, and is easy to transport, handle, and maintain (14). Elastic resistance also allows individuals to perform a range of ergonomic movements, increase or reduce the resistance or load using narrower or wider grips, and adjust the training intensity based on the rate of perceived exertion (15, 16).

In this context, the aim of this research was to assess adherence to a GFD, investigate the risk of suffering from an eating disorder, study blood nutritional variables, and determine the body composition in adult patients with CD undergoing different interventions of nutrition and physical activity.

## Materials and Methods

### Subjects

Twenty-eight women (57.21 ± 11.41 years), peri-menopausal and postmenopausal, participated in the study, 21 of whom were celiac patients. All of them were inhabitants of Alicante, Valencian Community, Spain. Each intervention group (4 groups) consisted of 7 women. The selection of the women was made among those who met the requirements of the study: peri-menopausal (≥ 60 days but < 1 year of amenorrhea) or postmenopausal (> 1 year of amenorrhea). For this, they were asked about regularity and their last menstrual cycle, in addition to the number of hot flashes they experienced throughout the day. The Celiac Association of the Valencian Community was contacted to help in the dissemination of the study. Exclusion criteria included having renal, thyroid, cardiovascular, and psychological diseases, or diabetes; using estrogen up to 3 months previously; following a specific diet; and having severe stress factors, such as the death of a close relative in the last month. First, an informative talk was given to inform them about the objectives, benefits, and commitment of the intervention. All women who were engaged in physical exercise or were consulting a nutritionist at the time of the study could not participate. The celiac women were eating a GFD in their own way, not planned by a specialized professional.

This study was approved by the University Human Research Ethics Committee of Alicante University (Spain) code UA-2018-10-22 and conducted per the guidelines laid out in the Declaration of Helsinki (17). All participants were informed about the study procedures and signed a written informed consent before entering the study. This trial was registered at clinicaltrials.gov as NCT05052164.

### Study Design and Interventions

**FIGURE 1:**
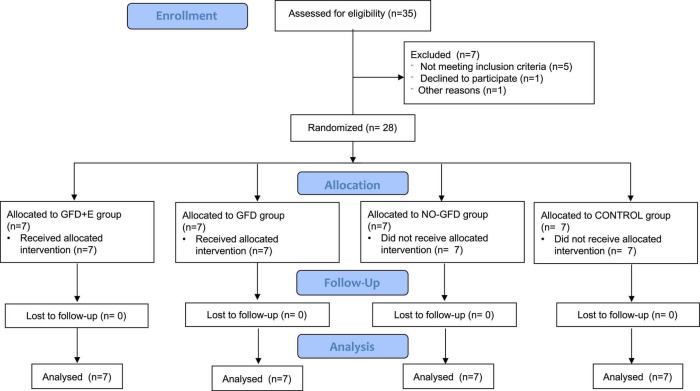
Consort 2010 flow diagram.

The study was designed as a 12-week randomized clinical trial. Eligible participants were randomized using 4-block randomization, with a separate randomization list using computer-generated random numbers. Once enrolled, subjects received the appropriate intervention: nutritional planning, resistance training, both interventions, or neither intervention, as shown in Figure 1. Group 1 (GFD + EF) consisted of 7 celiac women (44.7 ± 4.31 years) instructed by a dietician to consume a gluten-free isocaloric diet corresponding to their individual needs, determined by their specific resting energy expenditure (REE) which was calculated from the revised Harris–Benedict equation and adjusted for individual level of physical activity (18). In addition, they also performed personalized resistance training, led by a personal trainer with a degree in Physical Activity and Sport Sciences, following the current recommendations of the American College of Sports Medicine. The exercise intervention, which had been developed in a university clinic, began immediately after baseline. Resistance exercises were designed for all major muscle groups using elastic bands (Thera-Band^®^, The Hygenic Corporation, Akron, OH, United States). The sessions started with a warm-up consisting of aerobic exercises (15 min) for all of the muscle groups involved, followed by 30 min of resistance training based on elastic bands. The intensity of the exercises was progressively increased by adapting the resistance of the elastic band from yellow to red and further to black and by increasing the number of sets from one to two. All sessions (3 per week, for 12 weeks) were conducted and supervised by sport scientists. The perception of the training of each session was controlled with the Borg effort perception scale (1 up to 10). Group 2 (GFD) also consisted of 7 celiac women (56.3 ± 4.31) who followed a gluten-free isocaloric nutritional plan but did not exercise. Prior to the start of the research, women with celiac disease were following a GFD in their own way, not planned by a specialized professional. Group 3 (NO-GFD) consisted of 7 celiac women (62.4 ± 7.70) who did not receive any type of intervention, while group 4 (CONTROL) consisted of 7 healthy women (65.4 ± 4.12) who did not receive either nutritional planning or strength training. Daily physical activity was used as a control variable, for which all women wore a pedometer during the 12 weeks. The objective was to monitor daily physical activity.

### Measurements

#### Gluten-Free Diet Adherence

The Celiac Dietary Adherence Test (CDAT) (19) was used to value the adherence to a GFD. CDAT was created and validated by a panel of experts consisting of gastroenterologists, nutritionists, psychologists, and CD patients. It considers five of the most important aspects of adherence to a GFD the onset of CD symptoms, the patient’s knowledge of the disease and its treatment, confidence in the efficacy of the treatment, and motivating factors for adherence to a DLG, and self-reported adherence to the DLG. The questionnaire consists of 7 items on a 5-point Likert scale. The score ranges from 7 to 35 and is obtained by taking the sum of the numerical values assigned to the responses. The interpretation was as follows: 7 points—excellent adherence, 8–12 points—very good adherence, 13–17 points—insufficient/inadequate adherence, and > 17 points—poor adherence.

#### Psychological—Eating Disorders

Participants completed the Eating Attitudes Test (EAT-26). The EAT-26 is a commonly used self-reported measure (20) to determine whether there is a risk of developing an eating disorder. It is one used to indicate the level and type of symptomatology not for diagnosis. Participants provided their responses on a Likert-type scale of 6 frequency categories: always, almost always, frequently, sometimes, rarely, and never. The final score of the test corresponds to the sum of all items, which can vary between 0 and 78 points. The test items are structured based on three factors: diet, bulimia and preoccupation with food, and oral control. A score of 20 or higher was established as the cutoff value to identify women with possible eating disorders (ED) behaviors.

#### Physiological—Blood Samples

Fasting blood samples were obtained for the measurement of red cells, hemoglobin, hematocrit, mean corpuscular volume (MCV), mean corpuscular hemoglobin (MCH), mean corpuscular hemoglobin concentration (MCHC), leukocytes, platelets, neutrophils, eosinophils, lymphocytes, monocytes, basophils, glucose, total cholesterol, high-density lipoprotein cholesterol (HdL), low-density lipoprotein cholesterol (LdL), triglycerides, urea, creatinine, iron, and calcium. All variables were measured at the beginning of the intervention (W1) and at the end (W12).

#### Physical—Body Composition

At baseline (W1) and at the end of the study period (W12), body composition was assessed by anthropometry, following the standard protocol of the International Society for the Advancement of Kinanthropometry (ISAK). All measurements were performed by the same investigator, an ISAK level 2 anthropometrist. The mean technical error for perimeters, circumferences, lengths, and heights was less than 1% and for skinfolds less than 5%. All measurements were performed on the first day at the start of the intervention, under baseline conditions, in the same place, and at room temperature (22 ± 1°C).

Body weight (kg) was measured with a calibrated scale (BC545N, Tanita, Tokyo, Japan). Height was determined using a mobile anthropometer (Seca 213, SECA Deutschland, Hamburg, Germany) with millimeter accuracy, keeping the participant’s head in the Frankfort horizontal plane position.

All anthropometric equipment used was approved and previously calibrated: wall-mounted measuring rod (accuracy, 1 mm), digital scale (BC545N, Tanita, Tokyo, Japan; accuracy, 100 g), narrow, inextensible metal tape measure (Lufkin, TX, United States; accuracy, 1 mm), small bone diameter pachymeter (Smartmet, Jalisco, Mexico; accuracy, 1 mm), skinfold caliper (Harpenden, United Kingdom; accuracy, 1 mm), Supplementary Material (demographic pencil for marking celiac women), and a 40 × 50 × 30 cm anthropometric bench.

The 8 skinfolds (subscapular, tricipital, bicipital, iliac crest, supraspinal, abdominal, anterior thigh, and medial leg), 4 perimeters (relaxed arm, arm-contracted, thigh, and maximal leg), and 3 small bone diameters (biepicondylar of the humerus, bi-styloid, and bicondylar of the femur) were collected.

#### Statistical Analyses

Jamovi 1.1.3.0 software was used to perform all statistical analyses. Descriptive statistics (mean ± standard deviation) were calculated. The normality distribution was tested using the Shapiro–Wilk test. Baseline comparisons among groups were performed using one-way analysis of variance (ANOVA) followed by Tukey’s *post-hoc* test as appropriate. An ANCOVA (age used as a covariate) group × time was performed, followed by a Bonferroni *post-hoc* test to evaluate differences between the different times of the evaluations and treatments. For time × group interaction effects, partial eta squared effect sizes (η2) were calculated (η2 ≥ 0.01 indicates a small effect, ≥ 0.059 a medium effect, and ≥ 0.138 a high effect). Furthermore, to build connections between the study’s variables, the Pearson’s correlation test was performed with 95% confidence intervals. The statistical significance level was set at *p* ≤ 0.05.

## Results

### Baseline Characteristics

The baseline clinical characteristics of the study subjects are shown in Table 1. A total of 28 menopausal or postmenopausal women participated in the study. There were no significant differences between groups in the baseline variables.

**TABLE 1:** Baseline characteristics of the sample participating in the study.

	GFD + E	GFD	NO-GFD	Control
Age (years)	44.7 ± 4.31	56.3 ± 14.1	62.4 ± 7.70	65.4 ± 4.12
Height (cm)	166 ± 2.54	158 ± 1.79	159 ± 7.71	155 ± 4.50
BMI (Kg/m^2^)	26.2 ± 3.39	27.9 ± 3.67	24.6 ± 2.51	29.3 ± 4.43

*Data in the table are shown as mean ± SD. GFD + E, celiac women with a nutritional plan and physical exercise; GDF, celiac women with a nutritional plan; NO-GFD, celiac women with no nutritional plan or physical exercise; control, healthy women without diet or physical exercise intervention.*

### Adherence to a Gluten-Free Diet

**FIGURE 2:**
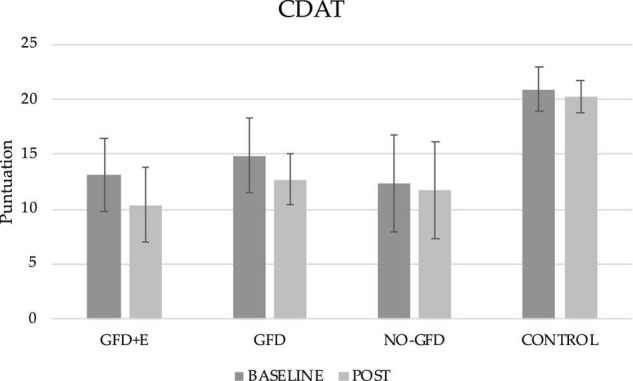
Descriptive statistics of the gluten-free diet adherence questionnaire before and after the intervention. CDAT, celiac dietary adherence test; GFD + E, celiac women with a nutritional plan and physical exercise; GDF, celiac women with a nutritional plan; NO-GFD, celiac women with no nutritional plan or physical exercise; control, healthy women without diet or physical exercise intervention.

Regarding adherence to a GFD, the results can be seen in Figure 2, both before and after the intervention. Significant differences have been observed in both the GFD + E and GFD groups. In the GFD + E group, adherence to a GFD increased (13.10 ± 3.29 vs. 10.40 ± 3.36; *p* < 0.001). In the GFD group, the score had decreased (14.90 ± 3.39 vs. 12.7 ± 2.36), therefore adherence had increased significantly (*p* < 0.001). In the group of celiac women who did not receive any intervention and the control group, no significant improvements were observed over time. After performing the *post hoc* analyses, significant differences were observed after the intervention between the GFD + E group and the control (10.40 ± 3.36 vs. 20.30 ± 1.50; *p* = 0.023), the GFD and control group (12.70 ± 2.36 vs. 20.30 ± 1.50; *p* = 0.016), and the NO-GFD group and control group (11.70 ± 4.42 vs. 20.30 ± 1.50; *p* = 0.001). In all cases, the adherence of the control group was much lower than in the rest of the groups.

### Eating Disorders

Table 2 shows the EAT-26 total score and subscale data for each group. Data are presented as mean ± standard deviation. No significant differences are observed in any group. Moreover, it is noteworthy that all women were at a fairly high risk for an eating disorder, as they all have a score > 20.

**TABLE 2:** Descriptive statistics of the different subscales of the EAT-26 in menopausal and postmenopausal women participants.

	GFD + E		GFD		NO-GFD		Control	
	Baseline	Post	Baseline	Post	Baseline	Post	Baseline	Post
EAT-26 TOT	44.0 ± 10.8	45.4 ± 11.1	47.3 ± 8.83	48.7 ± 7.39	50.1 ± 10.3	49.9 ± 13.8	41.1 ± 11.7	44.0 ± 11.9
EAT-26 Dieting	18.4 ± 6.50	19.3 ± 6.70	21.4 ± 6.63	22.0 ± 6.14	22.9 ± 7.47	22.7 ± 8.62	17.0 ± 5.89	19.1 ± 6.12
EAT-26 bulimia and food preoccupation	12.3 ± 2.87	12.3 ± 2.87	13.9 ± 2.12	14.1 ± 2.04	14.0 ± 2.71	13.6 ± 2.88	11.4 ± 3.05	11.6 ± 3.60
EAT-26 oral control	13.3 ± 2.29	13.9 ± 2.34	12.0 ± 4.55	12.6 ± 4.12	13.3 ± 4.19	13.6 ± 5.22	12.7 ± 3.30	13.3 ± 3.50

*EAT-26, eating attitudes test-26; mean differences were significant at p < 0.005; GFD + E = celiac women with a nutritional plan and physical exercise; GDF, celiac women with a nutritional plan; NO-GFD, celiac women with no nutritional plan or physical exercise; control, healthy women without diet or physical exercise intervention.*

### Blood Test Results

Table 3 presents the summary statistics of the ANCOVA analysis. In the leukocyte variable, significant differences were observed between the GFD and CONTROL groups (*p* = 0.004) after the intervention with the values being 5.06 ± 1.00 and 7.65 ± 1.44 microliters (μL), respectively, with the leukocyte level being lower in the GFD group. In addition, in leukocytes a trend (*p* = 0.051) was observed between the GFD + E and control groups, with a higher result in the control group. In the rest of the variables, as shown in Table 3, there were no significant differences.

**TABLE 3:** Effect of gluten free diet and resistance training (ANCOVA; the variable age has been included as a covariate).

	Effect time			Effect time × group		
	*F*1	*p*	η^2^	*F*1	*p*	η^2^
Red cells (%)	0.033	0.560	0.001	1.874	0.162	0.196
Hemoglobin (g/dL)	1.142	0.269	0.047	0.703	0.560	0.084
Hematocrit (%)	2.287	0.144	0.090	0.531	0.666	0.065
MCV (fL)	0.075	0.787	0.003	2.400	0.094	0.238
HCM (pg)	1.272	0.271	0.052	0.994	0.413	0.115
MCHC (g/dL)	0.357	0.556	0.015	0.360	0.782	0.045
Leukocytes (μg)	0.069	0.795	0.003	2.204	0.115	0.223
Platelets (μg)	1.104	0.304	0.046	0.890	0.461	0.104
Neutrophils (μg)	0.034	0.856	0.001	0.971	0.423	0.112
Eosinophils (μg)	0.588	0.451	0.025	0.753	0.532	0.089
Lymphocytes (μg)	0.725	0.403	0.031	0.305	0.822	0.038
Monocytes (μg)	2.840	0.105	0.110	2.500	0.085	0.246
Basophils (μg)	0.388	0.539	0.017	0.189	0.897	0.025
Glucose (mg/dL)	0.754	0.394	0.033	0.232	0.873	0.031
Cholesterol (mg/dL)	0.000	0.976	0.000	2.550	0.081	0.249
HdL cholesterol (mg/dL)	0.289	0.596	0.012	1.792	0.177	0.189
LDL cholesterol (mg/dL)	0.817	0.375	0.034	1.092	0.372	0.125
Triglycerides (mg/dL)	0.082	0.777	0.004	0.536	0.662	0.065
Urea (mg/dL)	0.010	0.919	0.000	0.285	0.835	0.036
Creatinine (mg/dL)	0.104	0.750	0.005	0.936	0.439	0.109
Iron (μg/dL)	0.802	0.308	0.034	2.215	0.114	0.224
Clacium (mg/dL)	0.463	0.503	0.020	0.290	0.832	0.036

*%, percentage; g, grams; dL, deciliter; μg, micrograms; mg, milligrams; fl, femtoliters; pg, pictograms. F1, effect; n^2^, eta squared (η^2^) effect sizes.*

### Anthropometry

**FIGURE 3:**
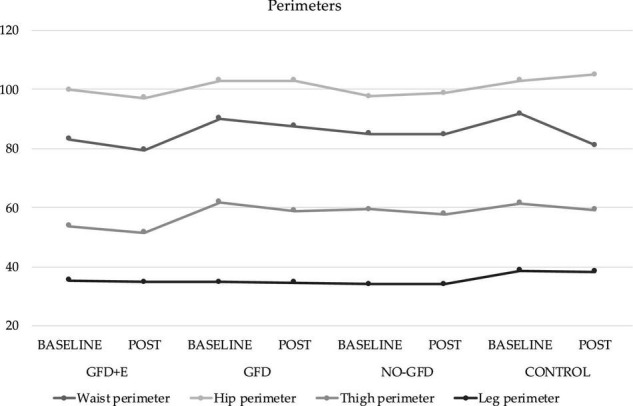
Descriptive statistics of perimeters before and after the intervention. GFD + E, celiac women with a nutritional plan and physical exercise; GDF, celiac women with a nutritional plan; NO-GFD, celiac women with no nutritional plan or physical exercise; control, healthy women without diet or physical exercise intervention.

**FIGURE 4:**
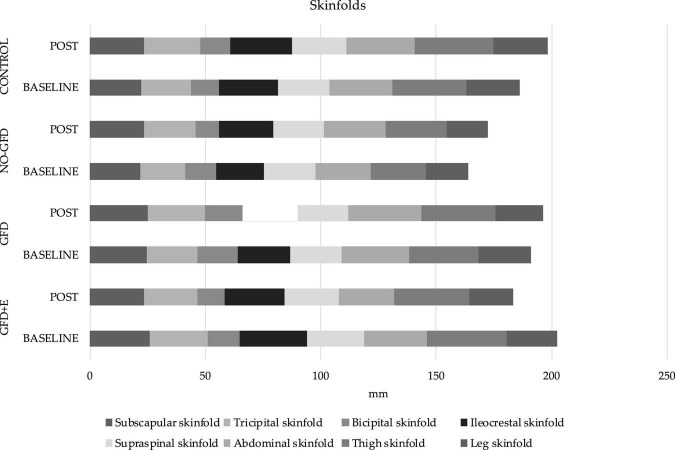
Descriptive statistics of skinfolds before and after the intervention. GFD + E, celiac women with a nutritional plan and physical exercise; GDF, celiac women with a nutritional plan; NO-GFD, celiac women with no nutritional plan or physical exercise; control, healthy women without diet or physical exercise intervention.

The anthropometric variables are presented in Figures 3, 4. In the ileocrestal skinfold, a significant trend was observed in the GFD + E group before (29.3 ± 3.21) and after (26.1 ± 5.34) the intervention (*p* = 0.053). There is also a significant time x group difference in the subscapular fold variable (*p* = 0.046); however, no differences can be observed in the *post hoc* analysis.

## Discussion

Adherence to a GFD is the main existing strategy to treat CD. In the present investigation, the groups that are formed by celiac women (GFD + E, GFD, and NO-GFD) presented a greater adherence after the intervention than at the initial assessment. At the beginning, the three groups had scores greater than 12, so adherence was insufficient. After the intervention, the scores decreased in the 3 treatment cases, therefore adherence to the GFD increased. However, the score was not less than 10 in any of the groups, indicating adherence to the GFD was good but not excellent.

Regarding physical activity, it has been observed that regular supervised physical exercises during a 16-week elastic band training intervention cause a significant improvement in body composition (fat mass loss) and lipid profiles in postmenopausal women (21, 22). This partially coincides with the results obtained, since the only group in which there was a decrease in all folds, and therefore fat mass, is the GFD + E group. Regarding the lipid profile, no significant decreases were observed. In the group doing physical exercise, the cholesterol levels have decreased from 194 ± 14.6 to 190 ± 27.6 mg/dL, HdL from 61.4 ± 15.0 to 56.6 ± 16.4, LDL from 117 ± 21.3 to 120 ± 30.2, and triglycerides from 78.4 ± 30.6 to 77.6 ± 41.7.

Adherence in the present study was measured using a standardized method, so the results obtained can be compared with others that have used the same tools. After data analysis, it has been shown that 33% of all celiac women did not adhere well enough to a GFD (score less than 13 points). Other investigations, such as Halmos et al. (23), evaluated the factors that may influence adherence to the GFD in patients with CD, using the CDAT questionnaire and other methods. Coinciding with the data obtained, they found that 61% of the patients adhered to a GFD. In 2016, Fueyo-Díaz et al. (24) reported after investigating a group of European participants with CD, in which 83.3% of the sample were women, that only 70% adhered perfectly or very well to a GFD. In another study carried out in the United Kingdom (UK), they stated (25) that only half of the patients in the UK (79% women, mean age 48 years) had sufficient compliance of a GFD according to the CDAT. Finally, confirming the above, a recent study performed by Gładyś et al. (26) observed that 48% of all patients (*n* = 92) had an excellent or very good adherence to a GFD. In addition to the prevalence of people with greater and lesser adherence to a GFD, in the present investigation, the result was compared with non-celiac women. The results obtained were as expected, 100% of the non-celiac women did not adhere to a GFD. After performing the correlations, it was observed that the patients with the highest CDAT scores had a higher body mass index (BMI) (*r* = 0.462; *p* = 0.013), therefore women with poorer adherence to the GFD had a higher BMI. It was also positively correlated (*r* = 0.384; *p* = 0.044) with leg or calf girth.

In addition to the classic gastrointestinal symptoms of CD, extraintestinal symptoms are increasingly recognized, such as neurological ones. Despite the current existence of research on the CD and psychiatric disorders, the literature is often contradictory (27). Regarding ED, it seems that there is a prevalence of ED in CD of 0.7%, observing that the probabilities of having an eating disorder are significantly higher in CD groups compared to controls (27). In the present investigation, the EAT-26 was used to assess the risk of suffering an ED, coinciding with Satherley et al. (6); higher EAT-26 scores were observed in CD compared to healthy controls. It appears that disordered eating behaviors are more prevalent in participants with chronic health conditions compared to healthy controls. The presence of binge eating behaviors in CD may be related to factors specific to the non-celiac disease, such as distress associated with the diet-controlled disease.

Serological variables were also analyzed in the present study. There were significant correlations between some clinical findings and body composition variables. Positive correlations were observed between the results of cholesterol and thigh circumference (*r* = 0.373; *p* = 0.050). The triglycerides variable also had a positive correlation with the hip circumference (*r* = 0.424; *p* = 0.025), thigh circumference (*r* = 0.517; *p* = 0.005), leg circumference (*r* = 0.476; *p* = 0.011), ileocrestal fold (*r* = 0.635; *p* < 0.001), supraspinal fold (*r* = 0.690; *p* < 0.001), and abdominal fold (*r* = 0.381; *p* < 0.045). Of the 22 serological parameters studied (red cells, hemoglobin, hematocrit, MCV, MCH, MCHC, leukocytes, platelets, neutrophils, eosinophils, lymphocytes, monocytes, basophils, glucose, total cholesterol, HdL cholesterol, LdL cholesterol, triglycerides, urea, creatinine, iron, and calcium), it is worth highlighting the significant difference that was observed between the leukocyte variable in the GFD group and the control group (*p* = 0.016) in the final evaluation, with the values being 5.06 ± 1.00 and 7.65 ± 1.44 μl, respectively. Leukocytes are white blood cells, which are part of the body’s immune system. At this point, it is important to consider that the gluten is made up of a group of proteins soluble in ethanol, that is, prolamins and glutelins, which are found in grains such as wheat (namely, gliadins and glutenins, respectively), rye (secalins and secalins), oats (avenins and avenalins), and barley (hordeins and herdenins). These proteins, rich in glutamine and proline residues, are resistant to the digestion of human intestinal proteases and provide elasticity to the mass necessary for expulsion and shaping (28). When gliadin peptides cross the epithelial lining and reach the bloodstream, they increase inflammation, thus spreading the immune response and causing extra-intestinal manifestations, including an increase in leukocytes, as has been proven in the present investigation.

In CD, there are often nutritional alterations, such as iron deficiency, anemia (3), or alterations in the lipidic profile (29), due to malabsorption. In women, the World Health Organization (WHO) defines anemia as having a blood hemoglobin concentration of less than 12 g/dl. Regarding iron levels in women, values between 50 and 140 μg/dl are established as normal (30). In previous research (7), it has been observed that 46% of CD patients had decreased iron storage and 32% had anemia. However, this does not coincide with the results obtained, since both the hemoglobin and iron data were normal, and none of the participants had values below the lower limit.

In relation to body composition, this is the first investigation where measurements of anthropometric variables have been carried out following the standard protocol of ISAK (31) in celiac women. Although the BMI is the most common indicator of adiposity in epidemiological research, it does not describe the distribution of body fat as an index of adiposity (32). Therefore, alternative indicators, such as skinfold thickness, have been explored, as it is an easy-to-use index for examining the torso and general obesity (33). It has been observed that people with a higher BMI (<27 kg/m^2^) also have a greater subscapular fold (34). In the present study population, the variable “subscapular fold” has increased in the GFD (24.9 ± 4.16 and 25.1 ± 6.36 mm), NO-GFD (22.0 ± 7.01 and 23.6 ± 7.72), and control (22.3 ± 7.7 and 23.6 ± 7.99) groups and decreased in the GFD + E (26.1 ± 5.52 and 23.3 ± 5.03) after the intervention. Although the differences are not significant, it seems that a GFD alone does not improve the thickness of some specific folds, such as the subscapular; however, the combination of diet and physical exercise does.

In addition to the previously described correlations between biochemical parameters (cholesterol and triglycerides) with different folds and perimeters, positive correlations are also observed between perimeters and the different EAT-26 scales. There are negative correlations between leg circumference, the EAT-26 total score (*r* = –0.457; *p* = 0.015), the diet control scale (*r* = –0.452; *p* = 0.016), and the bulimia scale (*r* = –0.496; *p* = 0.007). The same occurs with the supraspinal fold and the total score scales (*r* = –0.389; *p* = 0.041) and the control diet score (*r* = –0.403; *p* = 0.033). It seems that the greater the risk of suffering from CD, the less thick the skinfolds are.

It should be noted that in the present investigation 4 different groups could be compared (GFD + E, GFD, NO-GFD, and control); however, there were also several limitations to the study. These include the small sample size and the cost of the tests (despite having many serological variables). Finally, the methods to measure body composition, body folds, and perimeters were not ideal. Although this method is related to bone densitometry (double energy X-ray absorptiometry), it was not possible to use this method, which is considered the “gold standard,” due to its high economic cost and ease of access.

## Conclusion

It can be affirmed that people with celiac disease who have personalized nutritional planning according to their needs have a greater adherence to a GFD than those who do not have it planned by specialized professionals. This translates into a different immune system response since it has been seen that the control group (free gluten intake) presented significantly higher leukocyte values. A gluten-free isocaloric dietary plan is not enough to reduce body fat, since only the group that performed resistance sports showed improvements in anthropometric variables (skinfolds).

## Data Availability Statement

The raw data supporting the conclusions of this article will be made available by the authors, without undue reservation.

## Ethics Statement

The studies involving human participants were reviewed and approved by the Alicante University Ethical Committee. The patients/participants provided their written informed consent to participate in this study.

## Author Contributions

AM-R, JS-S, SP-M, and PM-P: conceptualization and resources. AM-R, PM-P, FA, and JR-A: methodology. AM-R and DL-M: software. JS-S, PM-P, and SP-M: validation. FA, RY-S, and JR-A: formal analysis. AM-R, JR-A, MM-O, NA-M, and DL-M: investigation. DL-M, FA, MM-O, RY-S, NA-M, and JR-A: data curation. AM-R, DL-M, JS-S, and MM-O: writing—original draft preparation. SP-M, FA, JR-A, RY-S, and PM-P: writing—reviewing and editing. AM-R and JR-A: visualization. AM-R and PM-P: supervision. AM-R: project administration. All authors have read and agreed to the published version of the manuscript.

## Conflict of Interest

The authors declare that the research was conducted in the absence of any commercial or financial relationships that could be construed as a potential conflict of interest.

## Publisher’s Note

All claims expressed in this article are solely those of the authors and do not necessarily represent those of their affiliated organizations, or those of the publisher, the editors and the reviewers. Any product that may be evaluated in this article, or claim that may be made by its manufacturer, is not guaranteed or endorsed by the publisher.
